# Dr. John Hutchens

**Published:** 1918-05

**Authors:** 


**Dr. John Hutchens,** Buffalo 1871, died at his home in Cheshire, N. Y., Feb. 23, aged 68.

